# Obesity and Low-Grade Inflammation Increase Plasma Follistatin-Like 3 in Humans

**DOI:** 10.1155/2014/364209

**Published:** 2014-07-03

**Authors:** Claus Brandt, Maria Pedersen, Anders Rinnov, Anne S. Andreasen, Kirsten Møller, Pernille Hojman, Bente K. Pedersen, Peter Plomgaard

**Affiliations:** ^1^Centre of Inflammation and Metabolism (CIM), The Centre for Physical Activity Research (CFAS), Department of Infectious Diseases, Rigshospitalet, University of Copenhagen, Blegdamsvej 9, 2100 Copenhagen, Denmark; ^2^Neurointensive Care Unit, Department of Neuroanaesthesiology, Rigshospitalet, Faculty of Health Sciences, University of Copenhagen, Blegdamsvej 9, 2100 Copenhagen, Denmark; ^3^Department of Clinical Biochemistry, Rigshospitalet, Blegdamsvej 9, 2100 Copenhagen, Denmark

## Abstract

*Background*. Rodent models suggest that follistatin-like 3 (fstl3) is associated with diabetes and obesity. In humans, plasma fstl3 is reduced with gestational diabetes.* In vitro*, TNF-*α* induces fstl3 secretion, which suggests a link to inflammation.* Objective*. To elucidate the association between plasma fstl3 and obesity, insulin resistance, and low-grade inflammation in humans.* Study Design*. Plasma fstl3 levels were determined in a cross-sectional study including three groups: patients with type 2 diabetes, impaired glucose tolerance, and healthy controls. In addition, lipopolysaccharide (LPS), TNF-*α*, or interleukin-6 (IL-6) as well as a hyperinsulinemic euglycemic clamp were used to examine if plasma fstl3 was acutely regulated in humans.* Results*. Plasma fstl3 was increased in obese subjects independent of glycemic state. Moreover, plasma fstl3 was positively correlated with fat mass, plasma leptin, fasting insulin, and HOMA B and negatively with HOMA S. Furthermore plasma fstl3 correlated positively with plasma TNF-*α* and IL-6 levels. Infusion of LPS and TNF-*α*, but not IL-6 and insulin, increased plasma fstl3 in humans.* Conclusion*. Plasma fstl3 is increased in obese subjects and associated with fat mass and low-grade inflammation. Furthermore, TNF-*α* increased plasma fstl3, suggesting that TNF-*α* is one of the inflammatory drivers of increased systemic levels of fstl3.

## 1. Introduction

Follistatin-like 3 (fstl3) is a circulating glycoprotein [1], which is highly expressed in the reproductive system, but fstl3 is also expressed in adipose tissue, pancreas, liver, and skeletal muscle [2, 3]. The only known function of fstl3 is to form high-affinity complexes with TGF-*β* family members, most notably activin A and myostatin and thereby inhibiting their function [4, 5]. Previous human as well as animal studies indicate that the regulation of these two target proteins is altered with obesity, diabetes or both. Thus elevated levels of activin A are found in adipose tissue from obese humans [6]. Moreover, patients with type 2 diabetes have elevated serum levels of activin A which correlate with markers of insulin resistance [7, 8]. Myostatin levels are elevated in serum from obese individuals [9] and patients with type 2 diabetes have elevated myostatin expression in the skeletal muscle tissue [10]. In prediabetic men, endurance training lowered myostatin in both serum and muscle [11].

In line with the impact of obesity and diabetes on activin and myostatin, reports from both human [12] and animal studies [13] suggest that fstl3 is involved in glucose metabolism and obesity. Thus, plasma fstl3 correlates inversely with the glucose concentration 1 hour after a 50 g oral glucose load in women whom later developed gestational diabetes. Additionally, low plasma fstl3 levels are associated with insulin resistance during pregnancy in humans and low plasma concentrations of fstl3 in the first trimester are associated with an increased risk of developing gestational diabetes [14]. In contrast, in obese leptin-deficient mice, fstl3 mRNA is increased in the subcutaneous adipose tissue [2] while fstl3 knockout mice have reduced visceral fat and hepatic steatosis [15]. Altogether, fstl3 could be involved in glucose metabolism and adipose tissue function.

Obesity and low-grade inflammation are tightly linked in humans [16, 17]. Inflammation may play a role in the regulation of fstl3 as the fstl3 promoter contains a NF-*κ*B binding site, and fstl3 protein is increased in response to TNF-*α* stimulation in HepG2 cells [18]. Interestingly, activin A, which fstl3 inhibits, is a part of the proinflammatory response [19].

The present study aimed at unravelling if circulating fstl3 is associated with and/or regulated by players in the metabolic syndrome, such as inflammatory mediators and insulin. Therefore, we studied fstl3 in a human cross-sectional study including healthy controls, individuals with impaired glucose tolerance, and patients with type 2 diabetes. Given that TNF-*α* stimulates fstl3* in vitro* and given the elevated levels of TNF-*α* in patients with obesity and insulin resistance [16], we measured fstl3 in response to* E. coli* lipopolysaccharide (LPS), TNF-*α*, and IL-6 infusions in humans. We hypothesized that fstl3 in humans would increase with insulin resistance and/or obesity and in response to inflammation. Here we demonstrate that fstl3 is elevated in obese subjects and that fstl3 increases in response to inflammation.

## 2. Materials and Methods

### 2.1. Cross-Sectional Study

Data from the cross-sectional part of this paper has previously been published [20] with data from 187 subjects. In the present study plasma fstl3 levels were measured in 200 subjects. The reason for the difference between the present and the previous papers with regard to* n*-value is that 13 persons were excluded in the first publication based on cognitive function. There is no data suggesting that fstl3 has an impact on cognitive function. Accordingly, we included all 200 subjects.

In brief, subjects who participated in the study were recruited to the cross-sectional study in order to obtain three groups representing different levels of glucose tolerance: a group with normal glucose tolerance (CON), a group with impaired glucose tolerance (IGT), and a group of subjects with type 2 diabetes mellitus (T2DM). The three groups were defined based on the oral glucose tolerance test (OGTT) according to WHO criteria. Insulin sensitivity and pancreatic beta-cell function were calculated by using the homeostatic model assessment (HOMA S and HOMA B) as described [28]. Obesity was defined as having a BMI of more than 30 kg/m^2^. OGTT, medication, VO_2max⁡_, and cytokine measurements were performed as described in [20]. Participants were allowed to take oral antidiabetic drugs but had to stop 1 week prior to the OGTT. After an overnight fast the participants reported to the laboratory for a general health examination; a blood sample was taken for measurement of fasting glucose, insulin C-peptide, TNF-*α*, IL-6, CRP, leptin, and adiponectin. This blood sample was also used for determination of fstl3. Dual-energy X-ray absorptiometry (DXA) was used to determine fat and lean mass as described in [20]. The participants were recruited by advertisement in local newspapers, and 3 subjects were recruited from the Steno Diabetes Centre database. Exclusion criteria were treatment with insulin, recent or ongoing infections, history of malignant cancer and severe chronic inflammatory diseases, fasting glucose above 12 mmol/L, and a blood pressure above 180 mm Hg/110 mm Hg.

### 2.2. Lipopolysaccharide (LPS) Bolus Injection

**Figure 1:**
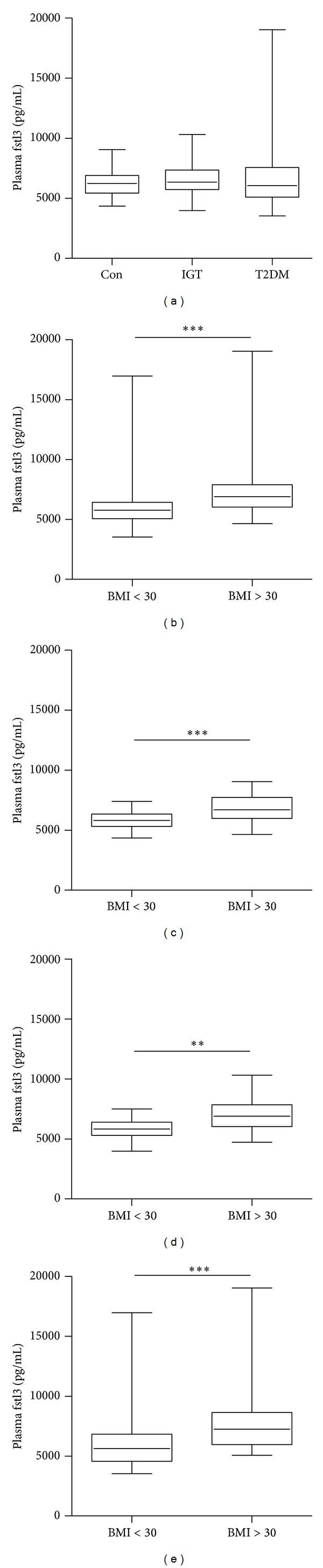
Plasma follistatin-like 3 in subjects with different glucose tolerance and in nonobese and obese subjects. (a) Plasma follistatin-like 3 (fstl3) subdivided by glycemic status (CON, *n* = 78), impaired glucose tolerance (IGT, *n* = 58), and patients with type 2 diabetes (T2DM, *n* = 64). (b) Plasma fstl3 in obese (*n* = 97) and nonobese subjects (*n* = 103). (c) Subdividing the control (obese = 36; nonobese = 42), (d) the impaired glucose tolerant (obese = 39; nonobese = 19), and (e) patients with type 2 diabetes (obese = 28; nonobese = 36). Boxplot showing median value of plasma fstl3 with 25th and 75th quartiles whiskers represents maximum and minimum value of fstl3. ∗∗ indicates *P* < 0.01. ∗∗∗ indicates *P* < 0.001.

Samples were retrieved from a study on the inflammatory response to LPS in T2DM compared to normal glucose tolerance [21–24]. Originally, the material included 23 healthy control subjects and 19 patients with type 2 diabetes [23]. Only samples from 12 healthy controls and 11 subjects with type 2 diabetes were available for the present study. Data on cytokine levels in response to LPS is shown in [23] (Figures 1(a)–1(c)).

In brief, subjects reported in the laboratory at 08:00 in the morning after an overnight fast and rested in the supine position while having catheters placed in antecubital veins for injection and blood sampling. An intravenous injection of* Escherichia coli* LPS (Lot G2 B274, United States Pharmacopeial Convention, Rockville, MD, USA) was administered at a dose of 0.3 ng/kg. Plasma fstl3 was measured at time points 0, 2 4, 6, and 8 hours.

### 2.3. TNF-*α* Infusion

The material has previously been presented [25, 26]. Originally the material included ten healthy male volunteers. Only samples from 8 volunteers were available for the present study.

In brief, subjects reported to the laboratory at 08:00 in the morning after an overnight fast and rested in the supine position while having catheters placed in antecubital veins for infusion and blood sampling [26]. Subjects received a 4-hour infusion of rhTNF-*α*(Beromun) 700 ng/m^2^·h^−1^ (Boehringer-Ingelheim, Biberach an der Riss, Germany) and on a separate day a 4-hour infusion of 20% albumin (TNF-*α* vehicle). The two study days were separated by at least one week. During the TNF-*α* infusion plasma TNF-*α* level was elevated to approximately 16 pg/mL and remained constant during the 4 hours of infusion as shown in [26] (Figure 1(a)). Fstl3 was measured at time points −2, 0, 1, 2, 3, and 4 hours.

### 2.4. IL-6 Infusion

The material has previously been presented [25, 27]. Six healthy male subjects participated in the study. In brief, subjects underwent a general health examination before participating [28]. Subjects reported in the laboratory at 08:00 in the morning after an overnight fast and rested in the supine position while having catheters placed in antecubital veins for infusion and blood sampling. Subjects received a 3-hour infusion of rhIL-6 (Sandoz, Basle, Switzerland) at a rate of 5 ug/h. During the infusion of rhIL-6 plasma IL-6 level was elevated to approximately 150 pg/mL as shown in [25] (Figure 1(a)). Fstl3 was measured at time points 0 h, 1 h, 2 h, 3 h, 6 h, and 24 h.

### 2.5. Hyperinsulinemic Euglycemic Clamp

Six healthy men aged 28.8 ± 2.5 years (mean ± SD) and BMI 22 ± 0.6 kg/m^2^ (mean ± SD) were recruited through local newspapers. Subjects underwent a general health examination before participating. The participants reported in the laboratory at 08:00 the morning after an overnight fast and rested in the supine position. Two peripheral intravenous catheters were placed: one for infusion of insulin and glucose and one for blood sampling. Subjects received continuous 5-hour infusion of insulin (Actrapid, 100 IU/mL; Novo Nordisk, Bagsvaerd, Denmark) at 0.05 U ∗ min^−1^ m^−2^. Blood glucose was measured at bedside and glucose was infused by a computer-controlled infusion pump at rates adjusted according to blood glucose levels in order to uphold euglycemia. Fstl3 was measured in plasma from samples at −15 minutes before the start of the clamp and at 45 min, 1 h 45 min, 2 h 45 min, 3 h 45 min, and 4 h 45 min relative to the start of the clamp.

All studies were approved by the ethical committee and performed in accordance with the Helsinki Declaration. Written consent was obtained from each subject prior to enrolment in the study.

### 2.6. Measurement of Plasma Follistatin-Like 3

In all studies, EDTA plasma was stored at −80°C. Plasma obtained after LPS and TNF-*α* study, fstl3 was measured using an fstl3 duo kit from cat number DY 1288 from R&D Systems. This kit has previously been used by others [12] and at the time of analysis the only available kit. The kit was used according to the manufacturer's instructions. In the cross-sectional study, the IL-6 infusion study, and the insulin clamp study, fstl3 was measured using a newer kit from Human FLRG (Quantikine ELISA Kit DFLRG0) from R&D Systems. Calculation of interassay variation was done by having two different control samples (in duplicate) on each plate. Mean value of fstl3 was calculated for each control, for the 6 plates used to measure fstl3 in the cross-sectional study. CV was 6.5% for control 1 and 7.4% for control 2. Intra-assay was calculated as the average CV for all the duplicates on each plate, by all 6 plates given a CV of 2.8% for the newest kit. Interassay variation CV% for the kit used in the LPS and the TNF-*α* studies was 3.8% calculated for three plates. Intra-assay variation calculated for the three plates used in the LPS study was 4.0%.

### 2.7. Leptin and Adiponectin Measurement

Plasma adiponectin, both high and low molecular forms, and leptin levels were measured using an ELISA kit from MSD (Meso Scale Discovery, Gaithersburg, USA) according to the manufacturer's instructions. Interassay variability was, 9% for leptin, and 17% for adiponectin, and intra-assay variability was 4.7% for leptin and 3.4% for adiponectin.

## 3. Statistics

As in the original cross-sectional study [20] data is presented as median and 25th + 75th quartiles. As many of the variables were not normally distributed, nonparametric tests were used for statistical analysis. To test for differences between the 3 glycemic groups, controls, IGT, and type 2 diabetics, Kruskall-Wallis test was used, and when significantly different, Wilcoxon rank test was applied to test CON versus IGT and CON versus T2DM corrected for multiple testing using Bonferroni correction. For differences between obese and nonobese, or between females and males, a Wilcoxon rank-sum test (two-sample) was applied. Correlations of fstl3 to different clinical markers were done using Spearman's correlations coefficient. All analyses on the cross-sectional study were done using SAS Software version 9.1 (SAS Institute).

In all infusion studies, plasma fstl3 is presented as geometric mean ± standard error of the mean (SEM). The effect of time and group on fstl3 by LPS and TNF-*α* infusion was assed using two-way ANOVA. The effect of IL-6 and hyperinsulinemia was assessed by one-way ANOVA using the Proc Mixed procedure, SAS 9.1. As a post hoc test a Tukey test was used. For all statistical tests, *P* < 0.05 was considered statistically significant.

## 4. Results

### 4.1. Subject Characteristics for the Cross-Sectional Study (Table 1)

As compared to healthy controls, patients with T2DM were older and had higher fasting insulin, glucose, C-peptide, and IL-6 levels, and lower adiponectin, HOMA S, HOMA B, and VO_2max⁡_. Subjects with IGT also had elevated fasting insulin, glucose, C-peptide, IL-6, and TNF-*α* levels and lower HOMA S and adiponectin levels compared to CON.

### 4.2. Plasma Fstl3 Is Elevated in Obese Subjects Independent of Glucose Tolerance (Figures 1(a)–1(e))

In the total study population of 200 subjects, median plasma fstl3 was 6244 (Q_25_ 5425–Q_75_ 7276) pg/mL. Fstl3 did not differ between CON (6216 (5447–6884) pg/mL), IGT (6344 (5733–7319) pg/mL), and T2DM groups (6062 (5111–7503) pg/mL). However, fstl3 was elevated in obese (BMI > 30 kg/m^2^) compared to nonobese subjects, both in the total group 6892 pg/mL (6031–7888 pg/mL) versus 5751 pg/mL (5081–6388 pg/mL) and within subgroups.

### 4.3. Plasma Fstl3 Is Correlated with Markers of Obesity, Insulin Resistance, and Inflammation (Tables 2 and 3)

In all subjects combined (*n* = 200), fstl3 correlated positively with age, weight (*P* < 0.001), hip (*P* < 0.001) and waist (*P* < 0.001) circumference, BMI (*P* < 0.001), whole body fat (*P* < 0.001), and trunk fat mass (*P* < 0.001) and negatively with height (*P* < 0.05) and VO_2max⁡_ (*P* < 0.001) (Table 2). Fstl3 also correlated with weight, hip and waist circumference, BMI, whole body fat, and trunk fat mass in individual groups. In the nonobese subjects, fstl3 was positively correlated with age, whole body fat, fat-free mass, and trunk fat. Plasma fstl3 correlated with age, hip and waist circumferences, BMI, whole body fat, trunk fat, fat-free mass lean body mass, and fat-free mass and negatively with VO_2max⁡_ in obese subjects.

Further analyses showed that plasma fstl3 was correlated with fasting plasma insulin, fasting C-peptide, and *β*-cell function assessed by HOMA B, but not with fasting glucose. Fstl3 correlated negatively with insulin sensitivity measured by HOMA S in all groups and in nonobese, but not in obese subjects.

In all subjects combined, plasma fstl3 correlated with plasma TNF-*α* and IL-6 (*P* < 0.001). In patients with T2DM, fstl3 correlated with both TNF-*α* and IL-6, whereas fstl3 only correlated with IL-6 and not TNF-*α* in the control and the IGT groups (Table 2). The association with TNF-*α* and IL-6 was present in both obese and nonobese subjects.

Fstl3 correlated with leptin in all subjects and subgroups. No correlation was observed to adiponectin.

### 4.4. Gender

Plasma fstl3 was higher in females than in males. Females have higher circulating fstl3 compared to males Plasma fstl3 was higher in females 6618 (Q_25_ 5769–Q_75_ 7429) pg/mL* n* = 100 than in males, 5892 (Q_25_ 5205–Q_75_ 6824 ) pg/mL* n* = 100; (*P* = 0.0024). This differences were only evident in obese (*P* = 0.002), but not between nonobese females and males (*P* = 0.083) (data not shown). As stated in Table 1 gender distribution did not differ between the different glycemic groups or was there a different in gender distribution between obese and nonobese subjects (*P* = 0.6712 Chi-square).

### 4.5. Plasma Fstl3 Increases in Response to Acute Inflammation (Figures 2(a)–2(c))

**Figure 2:**
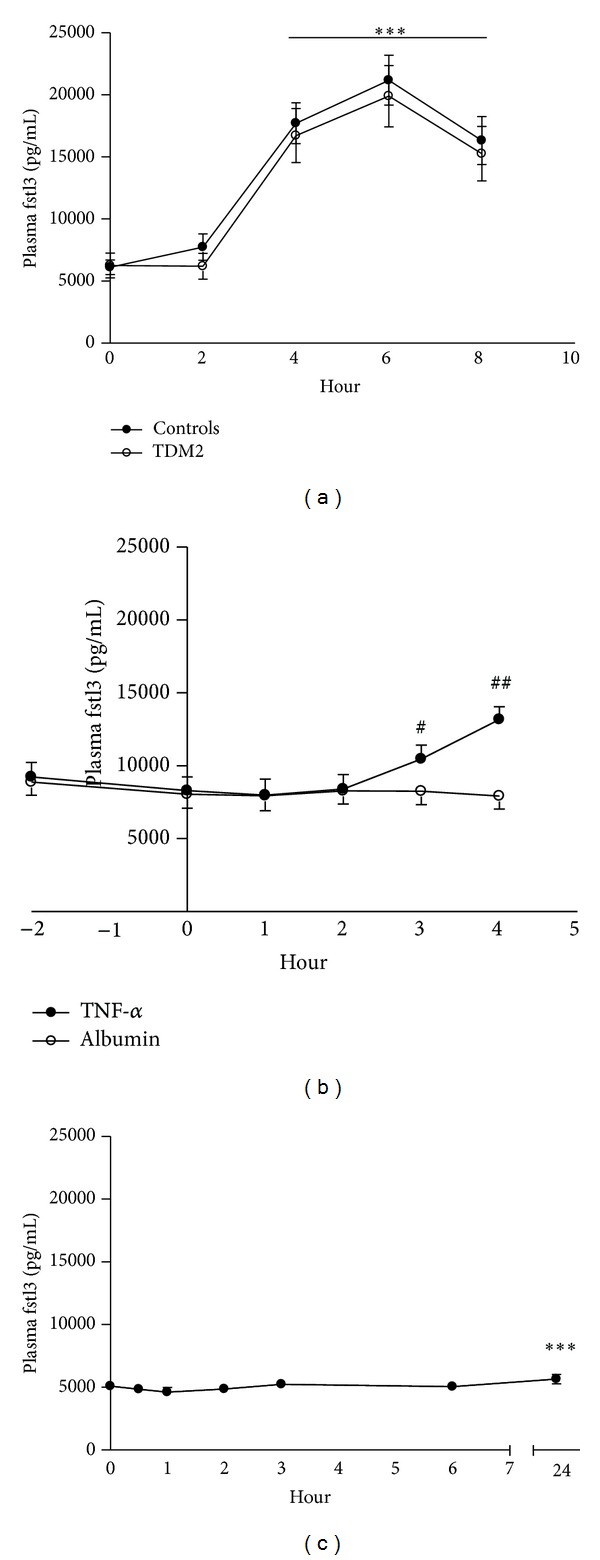
Plasma follistatin-like 3 in response to an LPS bolus and infusion of TNF-*α* and IL-6. (a) Effect of intravenous lipopolysaccharide (LPS) bolus (0.3 ng/kg) on circulating fstl3 in healthy subjects (*n* = 12) and patients with type 2 diabetes (*n* = 11) at prior to and 2, 4, 6, and 8 hours following the LPS bolus. No difference between the two groups existed, but a significant time effect. Data is presented as mean ± SEM. ∗∗∗ indicates a time effect *P* < 0.001, two-way ANOVA. (b) Effect of TNF-*α* infusion (700 ng/m^2^·h^−1^) on plasma fstl3. Two-way ANOVA: time *P* < 0.001, group *P* = 0.0012 and time ∗group *P* < 0.001. 2B. Data is presented as mean ± SEM (*n* = 8). ^#^Significantly different from albumin infusion *P* < 0.01. ^##^ *P* < 0.001. (c) The effect of IL-6 infusion (5 ug/h) on plasma fstl3. Data is presented as mean ± SEM (*n* = 6 ). ∗∗∗Significantly different from time points 0.5, 1, 2, 3, and 6 hours, one-way ANOVA.

As plasma fstl3 correlated with markers of low-grade inflammation, we investigated if plasma fstl3 was acutely regulated by inflammation. In the LPS study [21, 23], patients with type 2 diabetes and healthy controls were administered an LPS bolus of 0.3 ng/kg, which induced an inflammatory response with increased body temperature, heart rate, neutrophil count, and TNF-*α* and IL-6 levels [23]. In both groups, LPS increased circulating fstl3 over the 8-hour intervention period, with the highest levels observed after 6 hours. The response to LPS did not differ between controls and patients with T2DM (Figure 2(a)).

Next, we investigated if plasma fstl3 was regulated directly by the proinflammatory cytokines TNF-*α* or IL-6. During TNF-*α* infusion plasma fstl3 increased significantly after 3 hours and increased further at 4 hours (Figure 2(b)). No acute effect on plasma fstl3 was observed during IL-6 infusion (Figure 2(c)). However, the 24-hour time point was modestly but significantly elevated compared to all other time points.

### 4.6. Fstl3 in Response to a Hyperinsulinemic Euglycemic Clamp (Figure 3)

As fstl3 was strongly correlated to fasting insulin, we investigated the direct fstl3 response to a hyperinsulinemic euglycemic clamp. Plasma fstl3 was not changed in response to insulin.

## 5. Discussion

In the present study, the plasma level of fstl3 is elevated in obese subjects independent of glycemic status and is associated with fat mass as well as with plasma TNF-*α*, a strong marker of inflammation. Moreover, fstl3 is directly induced by both LPS and TNF-*α* administration, indicating that systemic inflammation increases fstl3 and that this effect is mediated by TNF-*α*. Finally, although plasma fstl3 is associated with markers of glucose intolerance, fstl3 is not increased in IGT or T2DM, suggesting an indirect rather than a direct relationship between insulin resistance and fstl3. A hyperinsulinemic clamp failed to increase fstl3 in plasma, suggesting that this putative relationship is not mediated by insulin.

Activin A is modestly increased in plasma from type 2 diabetic patients [7] and correlated with clinical parameter of type 2 diabetes [8]. In continuation, a 35% increase in serum myostatin is observed in obese humans [9]. Furthermore, training results in a 22% reduction in serum myostatin [11] in middle age men. Thus, the 20% increase in plasma fstl3 reported here in obese compared to nonobese subjects indicates that regulation of the myostatin/activin A fstl3 system in humans results in relatively small changes in circulating levels.

The few previous studies of fstl3 in humans have mainly addressed changes occurring during pregnancy. Thus elevated levels of fstl3 have been observed in women with preeclampsia [29, 30] and low levels of plasma fstl3 have been reported in women with gestational diabetes [12, 14, 29]. Fstl3 is highly expressed in the placenta [31]. During preeclampsia and gestational diabetes the expression of fstl3 in placenta is increased and decreased, respectively [14, 29, 30]. This suggests that the placenta may contribute to circulating fstl3 during pregnancy making comparison to type 2 diabetes and obesity difficult. In line with our data, others have demonstrated that fstl3 correlates with age and body weight [32] and BMI [12]. The observation that fstl3 correlated with age may be related to the increase in the ligands of fstl3, activin A and myostatin, which are increased with age [19, 33].

### 5.1. Fstl3 and Glucose Metabolism

In the present study plasma fstl3 is not different between controls, IGT, or T2DM and shows no association with fasting glucose suggesting that fstl3 is not involved in insulin resistance. Furthermore plasma fstl3 is not increased in response to insulin, indicating that fstl3 is not regulated by insulin. We did find a positive correlation between plasma fstl3 and fasting insulin, C-peptide and a negative correlation to insulin sensitivity (HOMA S). Low plasma fstl3 is associated with greater risk of developing gestational diabetes as fstl3 correlates negatively with peak glucose levels during a 50 g oral glucose load [12]. In another report also on gestational diabetes, no correlation to insulin resistance (HOMA IR) could be demonstrated with plasma fstl3 [14]. The difference between our data and the gestational diabetes studies may be explained by contribution of fstl3 to the circulation by the placenta during pregnancy. Furthermore, this could indicate that low fstl3 and thereby a potentially higher myostatin and activin A levels in plasma could be involved in the development of insulin resistance. In support of this view is the observation that activin A [34] but not myostatin [14] is increased in gestational diabetes.

### 5.2. Fstl3 and Adipose Tissue

In the present study plasma fstl3 is elevated in obese subjects and fstl3 is positively associated with total fat mass, trunk fat mass, and circulating leptin. Moreover, fstl3 is expressed in adipocytes and is increased in obese mice [2] and knockout of fstl3 in mice alters fat distribution [15]. Taken together this suggests that adipose tissue may contribute to circulating fstl3 levels. This is further supported by the finding that females, who generally have both higher fat percentage and leptin levels [35], also have higher fstl3 in plasma. As mentioned women with preeclampsia have elevated placental and plasma fstl3 levels [29, 30]. Leptin is also expressed by the placenta and plasma leptin levels are also increased in preeclampsia [36], which could indicate a common source or similar regulation of leptin and fstl3. Age is a possible confounder in the present study, as age is positively associated with plasma fstl3 in both nonobese and obese (Table 3). However nonobese subjects were older compared to the obese group (data not shown). This pattern was also present when the three glycemic groups were divided by obesity (data not shown). In the nonobese and obese groups 25 and 20 subjects were in treatment with statins, respectively. Therefore it is unlikely that plasma fstl3 is increased in the obesity group due to age or lipid lowering drugs.

### 5.3. Fstl3 and Inflammation

Exploring the association between fstl3 and low-grade inflammation observed in the cross-sectional study, by acute administration of LPS and TNF-*α*, we observed an increase of plasma fstl3 levels in humans. Activin A is well known for its acute proinflammatory actions as part of the innate immune system in response to LPS administration [37]. As fstl3 antagonizes activin A, the increase in fstl3 may potentially be considered a countercompensatory response to control the inflammatory process. This interpretation is consistent with the time course for plasma fstl3 in both the LPS and the TNF-*α* studies where fstl3 peaks later than activin A [37]. IL-6 does not acutely regulate fstl3 consistent with that IL-6 is not the driver for fstl3 during the LPS bolus. Fstl3 is increased after the IL-6 infusion at 24-hour time point compared with all other time points. The increase was modest before 5090 ± 227 versus 5657 ± 372 pg/mL at 24 hours. However, it is possible that IL-6 may increase fstl3 levels during chronic conditions such as obesity or low-grade inflammation [16].

### 5.4. Limitations

Due to the mainly cross-sectional design of the study, we cannot distinguish if obesity per se or low-grade inflammation is the main driver for the increased fstl3 in obese subjects. Neither are we able to clarify which tissue or tissues are responsible the circulating levels of fstl3 during obesity or inflammation.

## 6. Conclusion

We have demonstrated that circulating levels of fstl3 are increased in obese subjects and that high fstl3 levels are associated with fat mass and markers of low-grade inflammation. The finding that plasma fstl3 was increased in response to TNF-*α*, but not IL-6, infusion suggests that TNF-*α* is an inflammatory driver of increased systemic levels of fstl3.

## Figures and Tables

**Figure 3:**
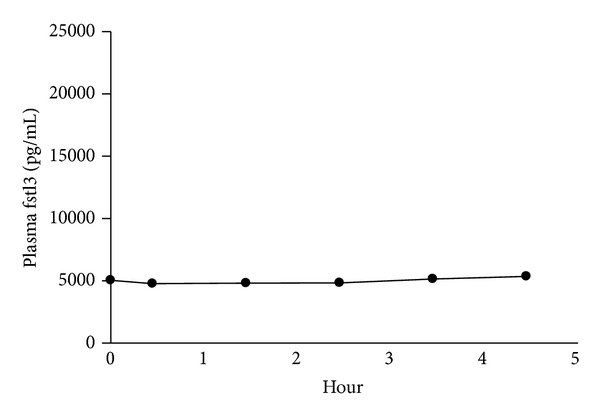
Plasma follistatin-like 3 during a hyperinsulinemic-euglycemic clamp. Plasma fstl3 during a hyperinsulinemic euglycemic clamp (0.05 U ∗ min^−1 ^m^−2^). Data is presented as mean ± SEM. Fstl3 was not altered by insulin (*P* = 0.12), one-way ANOVA (*n* = 6).

**Table 1:** Subjects' characteristics.

	Control (*n* = 78)	IGT (*n* = 58)	TD2M (*n* = 64)	One-way ANOVA
	Median (q_25_–q_75_)	Median (q_25_–q_75_)	Median (q_25_–q_75_)	
	42/36	32/26	26/38	0.189^$^
Age (years)	53 (48–60)	53 (47–60)	58 (51–62)∗	0.035
Weight (kg)	86.6 (74.7–109.3)	96.7 (80.6–114.1)	87.9 (79.9 103.4)	0.063
Height (m)	173 (164–182)	171 (164 182)	177 (166–181)	0.189
Hip (cm)	110 (101–118)	135 (105–124)	106 (100–114)	0.004
Waist (cm)	98 (89–115)	108 (97–122)∗	102 (96–110)	0.022
BMI (kg/m^2^)	29.4 (24.4–35.2)	32.0 (28–37)∗∗	28.6 (25.8–32.9)	0.003
Systolic BP (mmHg)	135 (126–150)	139 (130–153)	142 (132–151)	0.112
Diastolic BP (mmHg)	89 (79–86)	88 (84–98)	92 ( 85–97)	0.190
VO_2max_ (mL/min/kg)	26.0 (20.2–35.0)	25.1 (20.5–29.2)	24.0 ( 18.5–26.3)∗	0.029
Whole body fat (kg)	31.9 (20.4–40.6)	35 (29–45)∗	28 ( 21.5–37.1)	0.005
Trunk fat mass (kg)	17.1 (10.4–22.9)	20.5 (17.2–26.1)∗∗	17.3 (13.0–22.3)	0.003
Whole body fat-free mass (kg)	55.3 (42.4–64.1)	52.5 (44.3–65.3)	57.1 (45.9–65.9)	0.724
Insulin fasting (pM)	41.0 (21.0–57.0)	52.5 (36.0–88.0)∗∗	59.5 (30.0–102.5)∗∗	<0.001
Glucose fasting (mM)	5.5 (5.2–5.9)	6.1 (5.7–6.3)∗∗∗	8.4 (7.1–9.8)∗∗∗	<0.001
C-peptide (nM)	0.70 (0.53–0.89)	0.89 (0.70–1.18)∗∗∗	1.0 (0.69–1.25)∗∗∗	<0.001
HOMA B	57.3 (39.8–78.9)	62.1 (48.3–81.0)	31.4 (20.8–64.3)∗∗∗	<0.001
HOMA S	140.9 (89.6–249.1)	99.5 (60.1–159.5)∗∗∗	85.5 (52.5–160.7)∗∗∗	<0.001
TNF-*α* (pg/mL)	2.34 (2.04–2.82)	2.65 (2.25–3.04)∗	2.47 (2.12–3.45)	0.033
IL-6 (pg/mL)	1.51 (1.06–2.32)	2.01 (1.46–3.25)∗	2.33 (1.29–4.05)∗∗	0.007
Leptin (ng/mL)	10.8 (3.1–26.4)	15.3 (6.6–38.7)	7.8 (3.5–22.7)	0.033
Adiponectin (ug/mL)	15.4 (11.9–21.3)	12.2 (9.0–16.6)∗∗	10.2 (7.5–14.7)∗∗∗	<0.001

General description of the three glycemic groups. Data presented as median with 25 and 75 quartiles. The control group consisted of 78 subjects, the impaired glucose tolerance (IGT) of 58 subjects except for VO_2max_ (*n* = 57), whole body fat % (*n* = 57), and whole body fat-free mass (*n* = 57), and the patients with type 2 diabetes (T2DM) of 64 except for hip (*n* = 63), waist (*n* = 63), whole body fat % (*n* = 63), and whole body fat-free mass (*n* = 63). HOMA2 S: homeostasis model assessment of insulin sensitivity. HOMA B: homeostasis model assessment of beta-cell function. BMI: body mass index. *significantly different from the control group. *P < 0.05, **P < 0.01,  and ***P < 0.001. Corrected for multiple testing using bonferroni. ^$^Chi square test.

**Table 2:** Correlation between plasma follistatin-like 3 and demographic and biochemical characteristics.

	Total (*n* = 200)	Control (*n* = 78)	IGT (*n* = 58)	T2DM (*n* = 64)
	Spearman's *r*	Spearman's *r*	Spearman's *r*	Spearman's *r*
Age (years)	0.197∗∗	0.202	0.037	0.331∗∗
Weight (kg)	0.318∗∗∗	0.278∗	0.364∗∗	0.309∗
Height (m)	−0.178∗	−0.135	−0.162	−0.185
Hip (cm)	0.468∗∗∗	0.428∗∗∗	0.543∗∗∗	0.453∗∗∗
Waist (cm)	0.457∗∗∗	0.372∗∗∗	0.479∗∗∗	0.557∗∗∗
BMI (kg/m^2^)	0.477∗∗∗	0.420∗∗∗	0.547∗∗∗	0.447∗∗∗
Systolic BP (mmHg)	0.020	0.008	0.005	0.046
Diastolic BP (mmHg)	0.086	0.160	0.033	0.067
VO_2max_ (mL/min/kg)	−0.347∗∗∗	−0425∗∗∗	−0.146	−0.436∗∗∗
Whole body fat (kg)	0.585∗∗∗	0.560∗∗∗	0.598∗∗∗	0.624∗∗∗
Trunk fat mass (kg)	0.558∗∗∗	0.521∗∗∗	0.585∗∗∗	0.595∗∗∗
Whole body fat-free mass (kg)	−0.055	−0.058	0.073	−0.113
Insulin (fasting) (pM)	0.448∗∗∗	0.378∗∗∗	0.415∗∗	0.554∗∗∗
Glucose (fasting) (mM)	0.007	0.054	0.020	−0.071
c-peptide (nM)	0.453∗∗∗	0.409∗∗∗	0.380∗∗	0.598∗∗∗
HOMA B	0.372∗∗∗	0.398∗∗∗	0.371∗∗	0.455∗∗∗
HOMA S	−0.433∗∗∗	−0.368∗∗∗	−0.376∗∗	−0.566∗∗∗
TNF-*α* (pg/mL)	0.362∗∗∗	0.128	0.250	0.593∗∗∗
IL-6 (pg/mL)	0.497∗∗∗	0.371∗∗∗	0.363∗∗	0.695∗∗∗
Leptin (pg/mL)	0.598∗∗∗	0.614∗∗∗	0.545∗∗∗	0.629∗∗∗
Adiponectin (ug/mL)	0.044	−0.018	−0.040	0.176

Spearman's correlation coefficient is given for each variable in all subjects and in the different glycaemia groups. Spearman's correlation coefficient is given for each variable. The whole study group included 200 subjects except for hip (*n* = 199), waist (*n* = 199), BMI (*n* = 199), VO_2max⁡_ (*n* = 199), whole body fat (*n* = 198), whole body fat-free mass (*n* = 198). The control group consisted of 78 subjects, the IGT of 58 subjects except for VO_2max⁡_ (*n* = 57), whole body fat (*n* = 57), whole body fat-free mass (*n* = 57), and the T2DM of 64 except for hip (*n* = 63), waist (*n* = 63), whole body fat (*n* = 63), and whole body fat-free mass (*n* = 63). HOMA S: homeostasis model assessment of insulin sensitivity. HOMA B: homeostasis model assessment of beta cell function. BMI: body mass index. *indicates a significant correlation between plasma fstl3 and the given variable. *P < 0.05,  **P < 0.01, and ***P < 0.001.

**Table 3:** Associations between plasma follistatin-like 3 and biochemical and demographic markers in obese and nonobese subjects.

	Nonobese (BMI < 30) (*n* = 97)	Obese (BMI > 30) (*n* = 103)
	Spearman's *r*	Spearman's *r*
Age (years)	0.334∗∗∗	0.350∗∗∗
Weight (kg)	−0.091	−0.045
Height (m)	−0.164	−0.276∗∗
Hip (cm)	−0.002	0.376∗∗∗
Waist (cm)	0.079	0.207∗
BMI (kg/m^2^)	0.128	0.228∗
Systolic BP (mmHg)	−0.106	0.046
Diastolic BP (mmHg)	−0.108	0.087
VO_2max_/(mL/min/kg)	−0.149	−0.285∗∗
Whole body fat (kg)	0.312∗∗	0.466∗∗∗
Trunk fat mass (kg)	0.254∗	0.384∗∗
Whole body fat-free mass (kg)	−0.263∗	−0.311∗∗
Insulin fasting (pM)	0.292∗∗	0.195∗
Glucose fasting (mM)	−0.131	0.138
c-peptide (nM)	0.282∗∗	0.303∗∗
HOMA B	0.342∗∗∗	0.064
HOMA S	−0.217∗	−0.224∗
TNF-alpha (pg/mL)	0.334∗∗∗	0.273∗∗
IL-6 (pg/mL)	0.409∗∗∗	0.265∗∗
Leptin (pg/mL)	0.386∗∗∗	0.551∗∗∗
Adiponectin (ug/mL)	0.168	0.191

Spearman's correlation coefficient is given for each variable. The nonobese group included 97 subjects. The obese group consisted of 103 subjects except for hip (102), waist (102), VO_2max_ (102), and whole body fat (101). HOMA S: homeostasis model assessment of insulin sensitivity. HOMA B: homeostasis model assessment of beta cell function. BMI: body mass index *indicates a significant correlation between plasma fstl3 and the given variable. *P < 0.05,  **P < 0.01, and  ***P < 0.001.
